# A Comparative Study on the Influence of Psycho Social and Treatment Factors in Frequency of Episodes in Bipolar Affective Disorder

**DOI:** 10.1192/j.eurpsy.2023.1203

**Published:** 2023-07-19

**Authors:** M. I. Mundottu Kandy

**Affiliations:** Psychiatry, Tree Top Hospital, Republic of Maldives, Hulhumale, Maldives

## Abstract

**Introduction:**

Bipolar disorder is a chronic psychiatric illness of an episodic and recurrent nature with marked mood and behavioural dysfunction and causes substantial psychosocial morbidity, as it frequently affects independent living, vocational, and social activities. But there is a relative dearth of Indian research about the factors associated with risk of recurrence in patients with BPAD receiving treatment according to contemporary practice guidelines.

**Objectives:**

The study was under taken to assess the association of psycho-social and treatment factors with frequency of episodes in BPAD

**Methods:**

A cross-sectional study consisted of first 120 subjects with bipolar disorder who availed psychiatry services in a general hospital setting in central Kerala from January 2014 to July 2014. Diagnosis was made by DCR-10 criteria. Data for 114 subjects with BPAD were analyzed. Episode frequency was estimated as the number of episodes of depression, mania, and hypomania and mixed per year of illness. Stressful life events were assessed by Presumptive Stressful Life Event scale and treatment adherence by Drug Attitude Inventory. Modified Camberwell Family Interview were used for assessing expressed emotions and Kuppuswamy’s Socio Economic Scale for assessing SES

**Results:**

Episode frequency was significantly associated with young age group, female sex, low educational status, unemployment, lower socio-economic class, marital status, number of children, earlier age at onset, family history of BPAD, high stressful life events, high expressed emotions and poor treatment adherence. The association of co-morbid general medical condition and psychiatric condition with episode frequency were not significant. The influence of religion, family type and co-morbid substance use on episode frequency could not be commented

**Conclusions:**

Episode frequency was significantly associated psycho-social and treatment factors. Hence specific interventions are required to change the modifiable risk factors to reduce the recurrence in BPAD

**Disclosure of Interest:**

M. Mundottu Kandy Consultant of: NIL

